# Medical News and Miscellany

**Published:** 1899-01

**Authors:** 


### Medical News and Miscellany.

**Dr. P. A. Ramsel** has removed from Lincoln, Texas, to Rockdale, Texas.

---

**Dr. J. Wes. Carson** has removed from Indian Gap to Comanche, Texas.

---

**Dr. A. D. Nelson** has removed from Goldthwaite, Texas, to Big Valley, Texas.

---

**For Sale.**—One Gynecological Chair, in good condition; a bargain. Address, Dr. F. C. J., care of Texas Medical Journal.

---

**Spinal Meningitis** has made its appearance among the students of the Mississippi Agricultural and Mechanical College at

Starksville, Miss., and has caused a panic and a "scatteration" of the pupils.

---

**Married,** in San Antonio, Texas, January 4, ult., Dr. Richard Antonio Goeth, of Boerne, Texas, to Miss Lily Edith Dittman, of San Antonio.

---

**Mrs. Martha Hughes,** wife of Dr. Chas. H. Hughes, of St. Louis, died at her home on December 12, of paralysis of the heart brought on by the grippe.

---

**The "Red Back"** sends greeting to its readers, and wishes them all—and "their aunts and their uncles and their sisters and their cousins"—a Happy New Year!

---

**Erratum.**—In Dr. Jones' article, in third line from bottom on page 359, for "saturated with five per cent.," read "saturated with a five per cent. formaline solution."

---

**Married,** at Wiemar, Texas, December 21, 1898, J. Frank Thornton, A. M., M. D., of Plum, Texas, to Miss Marry E. Jarmon, daughter of R. A. Jarmon, Esq., of Weimar.

---

**Dr. Frank Rainey,** long-time superintendent State Blind Institute at Austin, has been appointed superintendent of the Widow's and Orphan's Home at Fort Worth, an institution founded by the Masonic fraternity of Texas. They couldn't have gotten a better man.

---

**The Meanest One Yet.**—Dr. H. H. Thorpe, of Liberty, Texas, attended the wife of a citizen in her confinement, presenting the fond husband with a fine boy. The doctor charged $10 for his fee, but "hubby" refused to pay it, and when four years later Dr. Thorpe sued for his fee the fellow pleaded "statute of limitation," and threw the costs on the good doctor. Shame!

---

**The Journal** calls special attention of its readers to the advertisement of "The *Alma*," in this issue. This is a grand sanitary institution delightfully environed, located at Alma, Michigan, just 4½ hours ride from Detroit or 8¼ hours from Chicago. It is a strictly scientific medical and surgical institution, offering unequaled attractions and advantages. It is conducted on a broad and liberal basis, and its management is loyal to and in harmony with the highest scientific practice of medicine and surgery. One would find it a luxury to be even sick at such a place; but, oh what a blessing it would be to convalesce and get well there! A handsomely illustrated little book descriptive of place and methods will be sent free on request.

---

**Hinc Illae Lacrimae.**—The Philadelphia *Medical Journal* has abolished the exchange-list, and notified all the journals that if they want the P. M. J. they will have to pay for it. Misery loves company; and the *Red Back* is somewhat consoled that it is not alone in this terrible calamity. It is pretty hard, but we will have to scrub along without it somehow. The Lord will provide.

---

**Apology.**—The *Red Back* for January is late, very late; but through no fault of the business management. It is due to de layed shipment, or rather non-arrival of paper from the factory. You fellows will be saying as the school-marm said to the boy who was late one morning, and gave as an excuse that his mother had had a baby: "Well, don't let it happen again." We won't.

---

**Wanted.**—Agents for "History of the Spanish-American War," by Henry Watterson. A complete, authentic history; illustrated with over 76 full-page half-tones and many richly colored pictures. Large royal octavo volume, superb outfit, postpaid for only 50 cents (stamps taken). Most liberal terms given. The greatest opportunity of the year. Address: The Werner Company, Akron, Ohio.

---

**It is stated** that nine of the leading homœopathic practitioners of Cleveland, O., have dropped the sectarian designation, and will hereafter be known as *physicians!* Let the good work go on until we have but one school of medicine.—*Medical Times.*

That's the idea. Those who call themselves homœpaths do not practice homœopathy; they are homos for revenue only. All honest homos ought to drop the sectarian title.

---

**Sewage Purification.**—The State Board of Health has approved plans for the purification of the sewage of East Cleveland. The sewage is to be purified by passing it through filters of coke, to which air will be constantly supplied by air pumping machin ery. The system is entirely new for Ohio, and the results, which are guaranteed, will be watched with much interest. Dr. A. B. Richardson, superintendent of the new State Hospital for the Insane at Massillon, reports that the purification of sewage by filtration just inaugurated there, is a marked success. The doctor is enthusiastic over the results.—*Ohio Bulletin.*

We can't have such luxuries in Texas; State "can't afford it."

---

**"Don't Use Alcohol."**—This may be good advice in more ways than one, but we mean it to apply to your hypodermatic syringe. Don't wash it out with alcohol; this dissolves the oil, which keeps the leather packing soft and pliable, and the latter consequently becomes hard and makes an imperfect joint. Wash out preferably with carbolated water. This will answer equally well as an antiseptic and will not shrink and dry the leather packing. Keep the cap on the syringe when not in use—this helps to prevent "drying out" of the packing.—*Therapeutic Notes.*

---

**Manufacture of Anti-Diphtheritic Serum.**—In our December number (ult.) we reproduced a portion of Dr. C. H. Powell's write-up of his visit to the laboratories of Parke, Davis & Co., at Detroit, where the manufacture of anti-diphtheritic serum is carried on under the strictest care of experts. By one of those unfortunate occurrences, which are said to take place even in the "best regulated families," (and publications also), the "copy" of our introductory to the abstract got badly mixed; a part of it being put at the end of the abstract instead of at the top. It was written as follows:

"Dr. C. H. Powell, of St. Louis, in the *North American Journal of Diagnosis and Practice*, for October, 1898, gives an interesting and instructive account of his visit to, and inspection of, the great laboratories of Parke, Davis & Co., Detroit. We abstract for the information of our readers the following remarks by Dr. Powell, on the methods adopted by this firm in manufacturing diphtheritic serum."

---

### Repeal of the Tax on Doctors.

AUSTIN, TEXAS, January 24, 1899.

*To the Physicians of Texas:*

We, your committee appointed at Dallas in December, 1897, have been at work all during the year and have done some good; and now is the time for final work. Our committee is now at Austin working with the Legislature for a repeal of the obnoxious occupation tax, and are greatly in need of funds to push same. Please remit to J. T. Wiggins, secretary, Austin, Texas, Box —, at once. The Legislature is favorable to our cause, but work must be done. Write your Senators and Representatives in behalf of the cause.

J. T. Wiggins, M. D., of Rusk,
Secretary State Central Committee.

